# Experiments upon the Gastric Digestion of Milk

**Published:** 1886-06

**Authors:** 


**Experiments Upon the Gastric Digestion of Milk.**—The following results were obtained by Reichmann in a serious of observations upon the length of time required for the digestion of milk in the healthy human stomach. About one half-pint of raw milk was usually given to the person under observation, and removed by the sound and pump at varying intervals of time. The quantity mentioned was found to be digested in three hours, and in four hours the last traces of acid fluid had left the stomach. The maximum gastric acidity (0.32 per cent.) was attained in one hour and fifteen minutes. Peptine appeared in noteworthy amount in from thirty minutes to two hours. Boiled milk was found to be somewhat more rapidly digested than was raw milk.—*Zietschr. f. klin. Med.*, Bd. ix. Hft. vi., 1885, p. 565.

